# Normalized tree water deficit: an automated dendrometer signal to quantify drought stress in trees

**DOI:** 10.1111/nph.70266

**Published:** 2025-06-11

**Authors:** Richard L. Peters, David Basler, Roman Zweifel, David N. Steger, Tobias Zhorzel, Cedric Zahnd, Günter Hoch, Ansgar Kahmen

**Affiliations:** ^1^ Tree Growth and Wood Physiology, TUM School of Life Sciences Technical University of Munich Hans‐Carl‐von‐Carlowitz‐Platz 2 Freising 85354 Germany; ^2^ Department of Environmental Sciences – Botany University of Basel Schönbeinstrasse 6 Basel CH‐4056 Switzerland; ^3^ Swiss Federal Institute for Forest Snow and Landscape Research WSL Zürcherstrasse 111 Birmensdorf 8903 Switzerland; ^4^ School of Biological Sciences University of Utah Salt Lake City UT 84112 USA

**Keywords:** maximum daily shrinkage, point dendrometer, stomatal conductance, temperate forests, tree water deficit, turgor loss point

## Abstract

- Trees often encounter periods of low soil water availability and high vapor pressure deficit, which induce drought stress and significantly impair their physiological functioning, growth, and survival. Automated dendrometers are valuable tools for quantifying signals related to tree water status, such as tree water deficit (TWD). Despite previous attempts, a robust method to clearly identify drought stress timing and intensity from TWD is still lacking.
- We established a novel normalized tree water deficit (TWD_norm_) index to quantify drought stress of trees that relies on TWD_norm_ and normalized maximum daily shrinkage (MDS_norm_). We validate our approach with measurements of leaf water potential, stomatal conductance (*g*_s_), and critical hydraulic thresholds from 118 trees from nine European tree species at the Swiss Canopy Crane II site.
- TWD_norm_ proved to be effective in identifying periods of decreased *g*_s_ and the onset of leaf turgor loss. We have found robust species‐specific thresholds for TWD_norm_ at which leaf turgor is lost, and the combination of TWD_norm_ and MDS_norm_ could be interpreted as a general index for drought stress.
- Deriving TWD_norm_ and MDS_norm_ solely from automated dendrometer data provides a unique biological drought stress signal that complements meteorological, hydrological, and satellite‐borne drought indicators.

## Introduction

Recent observations of forests under severe drought and heatwaves are alarming (Ault, 2020; Schuldt *et al*., 2020; Yuan *et al*., 2023; Bastos *et al*., 2024). Drought‐induced tree mortality has been linked to lethal embolisms in water transport systems (Choat *et al*., 2018; Arend *et al*., 2021) and turgor limitations causing growth reductions (Lockhart, 1965; Cabon *et al*., 2020; Peters *et al*., 2021). As drought events become more frequent (Dai, 2013; Vicente‐Serrano *et al*., 2020), forests' ability to store carbon and mitigate climate change may be significantly impaired (Hanewinkel *et al*., 2013; Pan *et al*., 2024). These concerns have driven efforts to monitor forest status and identify vulnerable species and hotspots of change (McDowell *et al*., 2015; Babst *et al*., 2021; Konings *et al*., 2021; Zweifel *et al*., 2023). Understanding critical thresholds, such as the leaf turgor loss point (Bartlett *et al*., 2012a, 2016) or embolism‐induced damage in xylem (Arend *et al*., 2021, 2022), is essential for assessing tree productivity and vitality.

Forest monitoring of tree growth is conducted through standardized forest inventories (Pretzsch *et al*., 2014), annual tree‐ring assessments (Klesse *et al*., 2020; Evans *et al*., 2024), monthly to weekly band dendrometer readings (Herrmann *et al*., 2016), or high‐resolution sub‐hourly automated dendrometers (King *et al*., 2013; Zweifel *et al*., 2016; Etzold *et al*., 2022). Automated dendrometers measure stem radius changes at the micron scale, capturing both growth and hydraulic signals driven by physiological processes occurring at the whole‐tree level (Zweifel *et al*., 2005, 2021; Steppe *et al*., 2006). Stem radius expansion occurs due to (nocturnal) tissue rehydration (mainly the phloem) and irreversible growth due to cambium cell division and expansion at full turgor. Stem radius shrinkage occurs due to diurnal tree water use when water demand for transpiration exceeds water supply by root water uptake and hydraulic conductivity (Steppe *et al*., 2006; Zweifel *et al*., 2016).

Beyond measuring growth, dendrometer data provides valuable insights into hydraulic signals, such as maximum daily shrinkage (MDS) or the daily amplitude of stem shrinking, calculated as the difference between the pre‐dawn maximum and midday minimum stem radius (King *et al*., 2013). MDS reflects the contribution of stored water in a tree to daily transpiration, as stem shrinking is proportional to water loss from the stem (Zweifel *et al*., 2000; McLaughlin *et al*., 2003). A smaller MDS relative to a given transpiration rate indicates a reduced capacity to buffer against atmospheric evaporative demand, signaling increased drought stress. However, interpreting MDS without explicitly knowing the transpiration rate, determined by atmospheric demand (vapor pressure deficit) and stomatal conductance (*g*_s_), can be inconclusive. For instance, MDS peaks at high transpiration rates but decreases when stomatal regulation occurs (i.e. reduced *g*_s_), particularly during periods of low water availability when rehydration is limited (Peters *et al*., 2023). Prolonged drought further reduces water storage pools, compounding the decline in MDS. Conversely, a reduction in MDS can also result from lower evaporative demand under conditions of high water availability, unrelated to drought stress. Developing new approaches to disentangle these contrasting MDS states would advance the creation of a more universal drought stress indicator.

Another valuable hydraulic signal derived from dendrometer readings is the tree water deficit (TWD), which quantifies stem shrinkage relative to the previous long‐term maximum stem radius (Zweifel *et al*., 2005). TWD is calculated using the zero‐growth concept, which assumes no irreversible stem size increase (e.g. bark or wood formation) occurs during shrinkage, meaning cambium cells are not at full turgor (Lockhart, 1965; Zweifel *et al*., 2016). Pre‐dawn TWD serves as a promising indicator of whole‐tree water status (Salomón *et al*., 2022; Steger *et al*., 2024), correlating effectively with leaf water potential (ψ_leaf_; Dietrich *et al*., 2018) and soil water potential (Walthert *et al*., 2021, 2024). As a key drought stress indicator, ψ_leaf_ reveals whether a tree species is approaching critical hydraulic thresholds (Steppe, 2018; Novick *et al*., 2022). However, TWD, like absolute MDS values, is highly tree‐ and species‐specific, making comparisons across sites, trees, and species challenging (Peters *et al*., 2023; Steger *et al*., 2024). This variability arises from differences in water storage tissue size, elasticity, and capacitance (Offenthaler *et al*., 2001; Salomón *et al*., 2017; Dietrich *et al*., 2018), though the underlying mechanisms remain poorly understood. Normalizing TWD using ψ_leaf_ measurements (Dietrich *et al*., 2018) or referencing long‐term stem radius trends (Zweifel *et al*., 2021) can improve comparability, but such approaches require extensive data over long periods, which is not always practical.

Peters *et al*. (2023) proposed a novel approach to interpret TWD by normalizing it with the maximum MDS (MDS_max_) observed across multiple vegetation seasons. This normalized metric, TWD_norm_, aims to reduce variability caused by interspecific and intraspecific differences in absolute shrinkage capacity. This approach allows for a more standardized comparison of stem water deficit across trees and species. Importantly, it builds on the idea that MDS_max_ reflects the overall capacity of stem tissues to shrink and can thus serve as a meaningful reference for interpreting stem radius changes of trees. By expressing MDS and TWD relative to MDS_max_ (i.e. as MDS_norm_ and TWD_norm_), it becomes possible to distinguish between short‐term (diurnal) fluctuations in water storage and long‐term water depletion in the stem. However, it remains unclear whether these normalized parameters can reliably serve as proxies for physiological performance, such as stomatal regulation or other hydraulic thresholds. For example, the relationship between *g*_s_ and MDS has not been systematically explored across species (but see Zweifel *et al*., 2007). Additionally, it is unclear whether a specific TWD_norm_ threshold can consistently signal turgor loss or hydraulic failure. Therefore, further evaluation is needed to determine whether MDS_norm_ and TWD_norm_ can meaningfully capture drought stress dynamics across tree species.

**Fig. 1:**
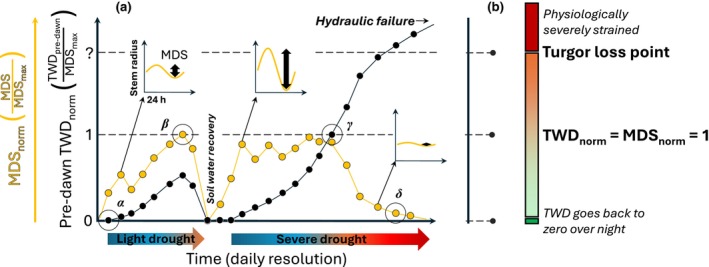
Schematic overview of physiological responses to pre‐dawn normalized tree water deficit (TWD_norm_) and normalized maximum daily shrinkage (MDS_norm_). (a) Time series over 24 d of daily pre‐dawn TWD_norm_ and MDS_norm_ spanning over a wide range of environmental drought conditions. The black circles highlight 3 d for which stem radius changes are shown in a higher temporal resolution and MDS is visualized with a bold black arrow. (b) Hypothetical interpretation of different relevant physiological thresholds is presented.

In this study, we evaluated the potential of combining TWD_norm_ with MDS_norm_ to develop easy‐to‐access measurements for drought stress. The conceptual Fig. 1 shows potential seasonal patterns of pre‐dawn TWD_norm_ and MDS_norm_ with some physiologically relevant thresholds. Along the axis of pre‐dawn TWD_norm_, drought stress is increasing, whereas along the MDS_norm_ axis, higher values also occur at relatively low TWD_norm_. The first physiologically relevant threshold, or point, is when pre‐dawn TWD_norm_ equals zero (Fig. 1, see *α*). This means that once within 24 h, TWD went back to zero, and the tree was able to fully rehydrate overnight. These are the days when trees can grow according to the zero‐growth concept (Zweifel *et al*., 2016). A second point is at MDS_norm_ = 1 (Fig. 1, see *β*). On such days, the tree shows daily shrinkage in the range of its all‐time MDS_max_, that is, high transpiration with no or little stomatal closure and no severe tissue dehydration that limits the shrinking process (pre‐dawn TWD_norm_ < 1). A third point is at pre‐dawn TWD_norm_ = 1 (Fig. 1, see γ). This point indicates the initiation of drought stress, as MDS_norm_ will afterward move below its maximum value (MDS_max_), where strong stomatal regulation and depleted storage tissues limit the daily stem shrinkage. At this stage, the tree is far away from being able to grow, and the overall physiological activity of the tree is reduced. During a continued severe drought (Fig. 1, see δ), more physiological activities are impacted, where the turgor loss point at a so far unknown value of TWD_norm_ > 1 should be reached. Above the turgor loss point, hydraulic failure and irreversible damage to cells, organs, or even the entire plant are very likely to occur.

To validate our methodological framework, we analysed data from 118 mature European trees across 9 species, collected from 2018 to 2023 at the Swiss Canopy Crane II (SCCII, Kahmen *et al*., 2022) site. We tested three hypotheses: H1 posits a negative relationship between *g*_s_ and pre‐dawn TWD_norm_, with a higher probability for stomatal closure after a TWD_norm_ surpasses 1. We expect this relationship with pre‐dawn TWD_norm_, as recent findings suggest that more negative pre‐dawn ψ_leaf_ conditions strongly impact *g*_s_ in mature trees (Peters *et al*., 2025). We assume this as there is a proven relationship between TWD and ψ_leaf_ (Dietrich *et al*., 2018). H2 suggests that trees reach their turgor loss point at a species‐specific threshold for pre‐dawn TWD_norm_ > 1. Here we assume that species crossing this threshold more frequently are more drought‐vulnerable, which is likely driven by the reduced conductance of the root–soil interface (Carminati & Javaux, 2020). H3 proposes that a species‐specific value of MDS_norm_ with increasing pre‐dawn TWD_norm_ can be used as a threshold to identify drought‐vulnerable species during drought periods without requiring ψ_leaf_ measurements (as done in H2), as species struggling to rehydrate tissues during low soil water availability will exhibit an earlier decline (Steppe *et al*., 2006). To test these hypotheses, we combined dendrometer measurements with bi‐weekly to monthly pre‐dawn and midday Ψ_leaf_ measurements, species‐specific turgor loss points, and xylem vulnerability curves (VCs) to assess hydraulic safety margins (HSMs). If we can confirm the hypotheses, these simple and easily obtainable dendrometer signals could serve as reliable indicators of whole‐tree drought stress in mature trees.

## Materials and Methods

### Site description and sampling design

Measurements were conducted at the SCCII site in Hölstein, Switzerland (47.439°N, 7.776°E, 500 m asl). Central to the site is a 50‐m‐tall canopy crane with a 62.5‐m jib, allowing access to the canopies of over 300 trees via a manned gondola. The site was installed in a mature mixed temperate forest on shallow clay‐rich soil over limestone. The 1.68 ha research area includes 476 living trees with a diameter at breast height (DBH) > 10 cm, representing 14 different species. The leaf area index is *c*. 2.2 m^2^ m^−2^, with a mean DBH of 31 cm (ranging from 10 to 80 cm, measured in 2017) and a mean tree height of 24 m (ranging from 4 to 36 m, measured in 2021). The site experiences a mean annual temperature of 9.6°C and an annual precipitation of 972 mm (1991–2020 average, MeteoSwiss climate station Rünenberg).

A total of 118 trees were monitored at the SCCII site using dendrometers from the start of 2018 until the end of 2023 (Table 1). The monitored species included the broadleaved *Fagus sylvatica* L., *Acer pseudoplatanus* L., *Fraxinus excelsior* L., *Carpinus betulus* L., and *Sorbus torminalis* Crantz, as well as conifers *Picea abies* Karst., *Abies alba* Mill., and *Pinus sylvestris* L. Hybrid *Quercus* trees, with varying degrees of *Quercus petraea* Liebl. and *Quercus robur* L., were treated as a single species for this study (*Quercus* sp.). The tallest trees within the crane's range from each of the nine species were selected as target trees. Target trees were occasionally replaced due to crown damage from drought or storm events or to avoid excessive pruning. Among these, 98 trees were monitored for *g*_s_ to track stomatal closure, and 115 trees were monitored for their Ψ_leaf_ dynamics as a proxy for drought stress. A total of 96 diurnal campaigns were conducted, typically from May to October, when leaves were fully developed. Each campaign included pre‐dawn sampling before sunrise (04:00 h–06:00 h CET) and midday sampling (12:00–14:00 h CET). During pre‐dawn sampling, Ψ_leaf_ measurements were taken, while both Ψ_leaf_ and *g*_s_ were recorded at midday. For each target tree, we measured the position, DBH, and tree height, using a Vertex IV (Haglöf, Sollefteå, Sweden).

**Table 1:** Overview of the tree size of the monitored dendrometer trees.

Species	DBH (cm)	Height (m)	Trees (*n*)
*Abies alba*	33.0 ± 13.8	25.9 ± 8.0	12
*Acer pseudoplatanus*	43.0 ± 12.5	28.8 ± 3.6	11
*Carpinus betulus*	26.7 ± 5.8	22.8 ± 4.7	11
*Fraxinus excelsior*	36.7 ± 4.2	31.0 ± 2.1	4
*Fagus sylvatica*	44.6 ± 9.4	29.6 ± 2.7	25
*Picea abies*	47.9 ± 10.3	31.9 ± 3.2	25
*Pinus sylvestris*	50.3 ± 7.1	32.0 ± 2.4	13
*Querucs* sp. (*Quercus robur* × petraea)	57.9 ± 13.2	31.0 ± 1.6	14
*Sorbus torminalis*	33.0 ± 8.9	22.9 ± 3.8	3

Besides the species, the diameter at breast height (DBH), total tree height, and the number of trees are provided. Mean values are shown with SD (±).

### Dendrometer installation and data processing

Point dendrometers (ZN11‐T‐WP, Natkon, Oetwil am See, Switzerland; Supporting Information Fig. S1) were installed on the target trees at breast height (1.3 m above the ground) on the north‐facing side of the tree stem. Before installation, the stem surface was prepared by carefully removing parts of the dead bark while avoiding damage to the underlying living bark. This ensured a smooth surface close to the phloem tissue and minimized hygroscopic swelling due to rainwater, following the guidelines of Zweifel *et al*. (2021). The dendrometers consisted of a carbon‐fibre frame, which was attached to the stem using three stainless steel threaded rods anchored *c*. 5 cm deep into the wood tissue. The metal sensing rod was gently placed on the prepared bark tissue to measure stem radius changes.

Data were collected using a DecentLab data logger (DecentLab GmbH, Dübendorf, Switzerland) and processed to a 10‐min resolution (example Fig. S1). The resolution of this dendrometer type was < 1 μm, with a temperature sensitivity of < 0.3 μm °C^−1^. All dendrometer readings were inspected for outliers using the datacleanr R package (Hurley *et al*., 2022) within the R software environment (v.4.2.2; R Core Team, 2022). After this inspection, one *Fagus sylvatica* dendrometer time series was discarded due to continuous shrinkage, likely caused by extensive crown dieback (Arend *et al*., 2022). The manually cleaned data were processed using the treenetproc R package (Knüsel *et al*., 2021) to extract TWD and maximum daily shrinkage (MDS).

Stem radius changes were partitioned into growth (irreversible radius increments) and water‐related components (reversible stem shrinkage and expansion) using the zero‐growth concept (Zweifel *et al*., 2016). This concept assumes that diameter variations below the preceding maximum stem radius are periods of TWD (in μm; Fig. S1). Additionally, MDS was extracted by analyzing daily stem radius dynamics and determining the difference between the pre‐dawn maximum stem radius and the minimum midday stem radius, provided such a shrinkage cycle occurred within a 24‐h period (see Knüsel *et al*., 2021 for details). Data were restricted to the period from May to October, when stem radius changes and related physiological processes are primarily influenced by transpiration and stem rehydration, rather than by phloem collapse in winter or temperature‐induced shrinking and swelling.

### Leaf water potential measurements

Leaf water potential measurements were conducted using a Scholander pressure chamber (PMS Instrument Company, Albany, OR, USA). We selected one to three small apical branches (5–10 cm long) with multiple healthy, sun‐exposed leaves or needles for placement in the pressure chamber. Leaf water potential was measured immediately after pre‐dawn and midday sampling with the canopy crane, recording the pressure in MPa at which water was expelled from the xylem tissue, while disregarding potential resin spillage in conifer species. For *Fraxinus excelsior*, instead of using small branches, we measured Ψ_leaf_ using the leaf and petiole, as the petiole xylem in this species is large enough to determine the point at which water is expelled from the xylem tissue, and this is less destructive to the tree.

### Stomatal conductance data

During the midday sampling campaigns, point measurements of *g*_s_ were taken from branches in the sunlit upper canopy using the crane gondola. We used a LI‐6800 Portable Photosynthesis System (LI‐COR Biosciences GmbH, Bad Homburg, Germany) equipped with either a broadleaf small light source chamber or a conifer chamber with a large light source. For broadleaved trees, healthy sun‐exposed leaves were selected, while for conifers, healthy sun‐exposed second‐year twigs were chosen to ensure fully developed leaves throughout the season. During measurements, the LI‐6800 was set to ambient temperature and relative humidity. The light source was adjusted to 1000 μmol m^−2^ s^−1^ with a 30% red and 70% blue ratio to mimic natural light conditions. All other system settings were configured according to the operational manual. For conifers, the leaf area was required to standardize the measurements obtained with the conifer chamber. One‐sided projected needle area was determined by spreading the needles from the measured twigs onto a flat‐bed scanner (Epson Expression 12000XL; Epson, Nagano, Japan). The needle area was then extracted from the scanned image using a dedicated digital analysis tool (github.com/dabasler/LeafAreaExtraction).

### Turgor loss point and vulnerability to embolism

In 2023, we collected branches from all target trees to measure the turgor loss points of each species. Throughout the growing season, samples were obtained from 78 trees using the canopy crane. Branches measuring 5–10 cm in length were harvested, stored in cooling boxes, and transported to the laboratory. Upon arrival, each branch was recut under water and rehydrated for a few hours by submerging the xylem in water, ensuring full rehydration by confirming that ψ_leaf_ was above –0.5 MPa (Arndt *et al*., 2015). From the fully hydrated branches, leaf disks (7 mm in diameter, using a cork borer) or needles were collected from fully developed leaves. The leaf material was then placed in small Eppendorf containers and stored in a −80°C freezer to fracture the membranes and walls. Subsequently, the Eppendorf containers were punctured with a needle and placed inside larger Eppendorf containers to extract the liquid via centrifugation. The osmolality of the collected liquid was measured using the OSMOMAT 3000 device (Gonotech, Berlin, Germany). Osmolality is expressed as molar concentrations of solutes per mass of water (mol kg^−1^). Turgor loss point was estimated following Sjöman *et al*. (2015), based on Bartlett *et al*. (2012a,b), by converting leaf osmolality to osmotic potential and applying an empirical relationship. This turgor loss point serves as a crucial ecophysiological threshold for a heavily drought‐stressed plant.

Embolism vulnerability was assessed to calculate the second crucial threshold, namely the lethality of drought stress experienced by the trees. This vulnerability was used to calculate the HSM (Hartmann *et al*., 2021). Vulnerability curves were obtained from Kahmen *et al*. (2022), who performed these measurements at the same research site as this study. In brief, samples for the VCs were collected from the target trees at the SCCII site during various sampling campaigns in 2019 and 2022. Branches *c*. 100 cm in length were harvested from the upper, sun‐exposed canopy. After collection, the samples were transported to the University of Würzburg, where VCs were established using the flow centrifuge method (Cochard *et al*., 2005) with a custom‐made rotor (Delzon *et al*., 2010) attached to a Sorvall RC‐5C centrifuge (Thermo Fisher Scientific, Waltham, MA, USA). The pressure at which 50% loss of conductivity was reached was used in this study (see Kahmen *et al*., 2022 for details). For *S. torminalis* and *Quercus* sp., we used data from the Botanical Garden in Würzburg or a nearby field site (Dietrich *et al*., 2019), while for *F. excelsior*, we used the value from ring‐porous *Quercus* sp. as a surrogate.

### Data analyses and statistics

Data processing and statistical analyses were conducted using the R programming language. Linear mixed‐effect models (LMMs) were executed with the lme4 package (Bates *et al*., 2015), and *post hoc* tests were performed using the emmeans package (Lenth *et al*., 2018). Model selection involved including or excluding interactions or variables based on the Akaike information criterion (AIC) and logical reasoning. The models presented in this manuscript were tested for assumptions by systematically inspecting for common issues as outlined by Zuur *et al*. (2010): (1) outliers, (2) homogeneity, (3) normality, (4) zero trouble, (5) collinearity, (6) relationship, (7) interactions, and (8) independence. For describing average patterns of processes that do not exhibit clear linear behavior, generalized additive mixed models (GAMMs) were used with the gamm4 and mgcv packages (Wood, 2020).

Normalized TWD (TWD_norm_) was calculated as the pre‐dawn TWD (TWD_pre‐dawn_) divided by the maximum MDS during the vegetation period (MDS_max_), determined as the 99^th^ percentile of all MDS values over a multi‐year period (Eqn 1). Using TWD_pre‐dawn_ was suggested by Salomón *et al*. (2022) and reflects the minimal TWD within 24 h. Here, we evaluate the potential of combining TWD_norm_ with normalized maximum daily shrinkage (MDS_norm_; Eqn 2) as an indicator for drought stress.

(Eqn 1) $$\mathrm{Pre}‐\text{dawn}\;{\mathrm{TWD}}_{\mathrm{norm}}=\frac{{\mathrm{TWD}}_{\mathrm{pre}‐\text{dawn}}\;}{{\mathrm{MDS}}_{\max}}$$

(Eqn 2) $${\mathrm{MDS}}_{\mathrm{norm}}=\frac{\mathrm{MDS}}{{\mathrm{MDS}}_{\max}}$$

To test our hypotheses, we used TWD_norm_ and MDS_norm_ for each tree‐specific time series. Both parameters were scaled (or *z*‐scored; mean = 0 and SD = 1) to make it possible to compare the response strength between these two independent variables. Critical for our hypotheses is the establishment of the robustness of determining MDS_max_, as this directly affects TWD_norm_ and MDS_norm_. Therefore, we performed an annual random bootstrap resampling analysis to test how many years are sufficient for obtaining stable MDS_max_ values across species (equivalent to the MDS_max_ established for our 6‐yr‐long time series). For H1, we investigated whether TWD_norm_ and MDS_norm_ reduced *g*_s_. An LMM was fitted across species with the TWD_norm_ and MDS_norm_ as interacting explanatory variables for *g*_s_, with tree nested within species as random effect. Stomatal conductance values below zero were removed, and the remaining values were log‐transformed to ensure a normal distribution of residuals. A total of 13 data points from the original 950 were excluded as they were below 0 mol m^−2^ s^−1^ *g*_s_. Due to the presence of first‐ and second‐order autocorrelation, autoregressive modelling was incorporated.

For H2, we tested whether a species‐specific TWD_norm_ exists at which species reach critical water potential thresholds, including the turgor loss point and the point of 50% xylem cavitation (*P*_50_). We analyzed this by relating ψ_leaf_ to TWD_norm_ and checked at which point the measurements crossed the ecophysiological thresholds. An LMM was fitted across species with ψ_leaf_ as the explanatory variable for pre‐dawn TWD_norm_, with tree nested within species as random effect. For this analysis, solely ψ_leaf_ values between −0.5 MPa and the species‐specific *P*_50_ point were used. This was done to ensure a linear model fit, as points outside of these ranges show a nonlinear behavior.

For addressing H3, we tested whether a specific threshold of MDS_norm_ at higher TWD_norm_ could serve as an indicator of drought stress in tree species. We used a boundary‐line analysis (Peters *et al*., 2019) to relate TWD_norm_ (pre‐dawn and midday) to MDS_norm_, focusing on maximum values within specific x‐ranges. This method accounts for days when transpiration is suppressed by low vapor pressure deficit or cloudiness. TWD_norm_ was binned into 0.2 increments, and maximum MDS_norm_ values were extracted for each tree‐specific time series. The bin size was chosen to balance the number of bins with sufficient data points per bin. GAMMs were used to assess the maximum MDS_norm_ response to TWD_norm_, and the TWD_norm_ value at 50% MDS_norm_ (MDS_norm_ = 0.5) was identified as a critical threshold. Note that we selected the maximum value of MDS_norm_ per tree with increasing TWD_norm_ bin values, as MDS_norm_ can also pass the 50% threshold at low TWD_norm_ values due to low atmospheric demand for water (i.e. at low vapor pressure deficit). This 50% threshold was selected because it is commonly used in ecophysiology, such as ψ_gs50_ (Anderegg *et al*., 2017), and it is easy to interpret. To validate these thresholds, we compared the species rankings derived from TWD_norm_ thresholds with rankings based on HSMs, calculated as the difference between the minimum ψ_leaf_ per tree and the ψ_leaf_ at which 50% xylem cavitation occurs. Due to the (bi)weekly ψ_leaf_ measurement frequency, we could not confirm whether the minimum midday ψ_leaf_ values for 2023 were captured, unlike with the daily dendrometer data, limiting direct comparisons of individual trees' HSM.

## Results

### H1: Relationship between TWD_norm_ , MDS_norm_ , and *g*_s_

**Fig. 2:**
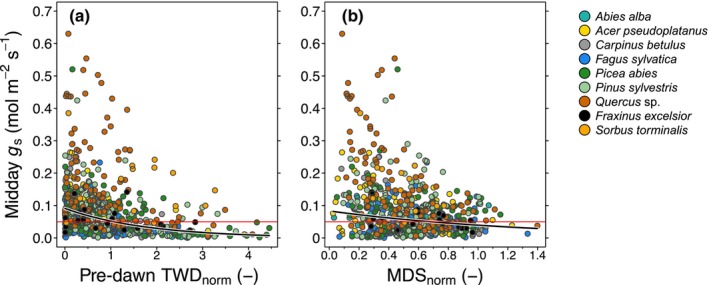
Relationship between pre‐dawn normalized tree water deficit (TWD_norm_), maximum daily shrinkage (MDS_norm_), and midday stomatal conductance (*g*_s_) over all species. A linear mixed‐effect model was fitted on the data (random effect = random intercept of tree nested in species), with the solid black line showing the mean. Within the model, (a) pre‐dawn TWD_norm_ and (b) MDS_norm_ are interacting independent variables to explain midday *g*_s_. The colors correspond to different species, as shown in the legend. Red lines indicate the 0.05 mol m^−2^ s^−1^ *g*_s_ threshold, which is added to illustrate highly constrained *g*_s_ (Xu *et al*., 2022). Note that MDS_norm_ can be above the value of 1 as maximum MDS (MDS_max_) is determined as the 99^th^ percentile of the MDS time series. Analyses include the following tree species: *Abies alba*, *Acer pseudoplatanus*, *Carpinus betulus*, *Fagus sylvatica*, *Picea abies*, *Pinus sylvestris*, *Quercus* sp., *Fraxinus excelsior*, and *Sorbus torminalis*.

TWD_norm_ and MDS_norm_ exhibited a significant negative relationship with midday *g*_s_ (conditional *R* ^2^ = 0.55; Fig. 2; Table S1). Model selection using the AIC indicated that the relationship was best explained by log‐transforming *g*_s_. Moreover, the scaled slope for TWD_norm_ was −0.48, while the scaled slope for MDS_norm_ was only –0.18 (Table S1), indicating that the impact of MDS_norm_ on *g*_s_ was weaker (Fig. 2b). These findings confirm H1, as it appears that TWD_norm_ relates well to midday *g*_s_ (Fig. 2a). The same relationship was observed when normalizing *g*_s_ to the species‐specific maximum to reduce absolute variance between species, and not considering the interaction (see Fig. S2; *P* < 0.001, conditional *R* ^2^ = 0.23). When calculating TWD_norm_ (Eqn 1), we observed that the mean behavior across species crossed the stomatal closure threshold at TWD_norm_ of *c*. 1 (Fig. S3). Our bootstrap resampling analysis revealed that a time series of MDS longer than 2–3 yr generated MDS_max_ values equivalent to our 6‐yr‐long MDS time series (Fig. S4), confirming the robustness of our MDS_max_ estimations.

### H2: Species‐specific thresholds of turgor loss at a TWD_norm_ above 1

**Fig. 3:**
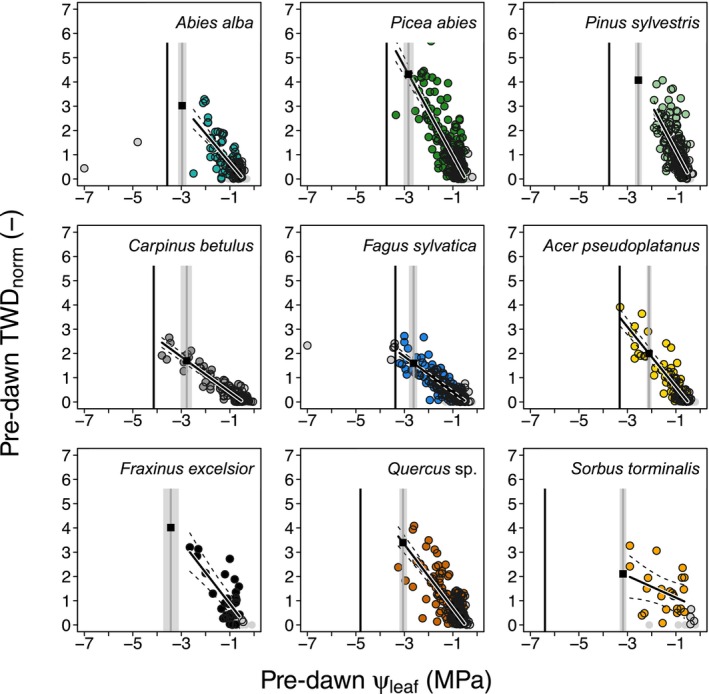
Relationship between pre‐dawn leaf water potential (Ψ_leaf_) and pre‐dawn normalized tree water deficit (TWD_norm_). Critical ecophysiological thresholds presented include the turgor loss point (solid gray line with the upper and lower quantile as shading) and the point of 50% loss of xylem conductivity (vertical black line). Zero TWD_norm_ values are indicated by gray dots. Also, Ψ_leaf_ values above −0.5 MPa and below the point of 50% loss of xylem conductivity are presented as gray circles/dots and not included in the analysis to ensure linearity. A linear mixed‐effect model was fitted to illustrate the species‐specific behavior (bold black line), including the confidence interval (dashed lines). Analyses include the following tree species: *Abies alba*, *Acer pseudoplatanus*, *Carpinus betulus*, *Fagus sylvatica*, *Picea abies*, *Pinus sylvestris*, *Quercus* sp., *Fraxinus excelsior*, and *Sorbus torminalis*.

The physiological relevance of pre‐dawn TWD_norm_ was tested by relating it to pre‐dawn Ψ_leaf_ and comparing it to known ecophysiological thresholds (Fig. 3). Note that Fig. 3 only includes days at which we have measured ψ_leaf_, while for H3, we have more data points, as we have continuous daily measurements. We found strong linear relationships between ψ_leaf_ and pre‐dawn TWD_norm_ for most species (weaker for *S. torminalis*, due to the low number of data points). We detected species‐specific pre‐dawn TWD_norm_ values at which ψ_leaf_ values were reached, where we assume leaf turgor loss. The results indicate that the species *A*. *alba*, *P. abies*, *C. betulus*, *F. sylvatica*, and *A. pseudoplatanus* experienced periods where their turgor loss points were crossed. By contrast, *P. sylvestris*, *F. excelsior*, *Quercus* sp., and *S. torminalis* exhibited less drought stress. A similar drought stress ranking was observed when considering midday ψ_leaf_ values (Fig. S5), demonstrating that pre‐dawn and midday ψ_leaf_ converge at the point of turgor loss. Only *A. alba*, *P. abies*, and *F. sylvatica* clearly crossed the hydraulic threshold at which 50% of hydraulic conductivity would be lost (*P*_50_), either during pre‐dawn (Fig. 3) or during midday ψ_leaf_ conditions (Fig. S5).

### H3: Drought sensitivity and its ranking based on HSMs

**Fig. 4:**
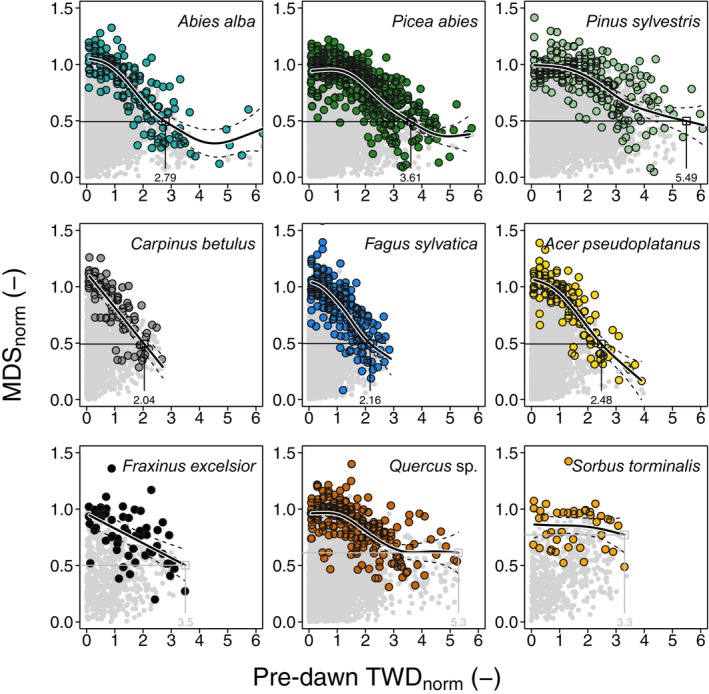
Species‐specific response of normalized maximum daily shrinkage (MDS_norm_) to pre‐dawn normalized tree water deficit (TWD_norm_). Maximum values across fixed‐size TWD_norm_ bins were determined for each tree (colored dots) from the raw measurements (gray dots). A generalized additive mixed model (GAMM) was fitted to depict the species‐specific behavior (bold black line), with the confidence interval shown by dashed lines. The number in each panel represents the point at a MDS_norm_ of 0.5, which provides an idea of the steepness of the relationship. If a normalized MDS of 0.5 was never reached by the GAMM, the maximum value is presented in gray (i.e. for *Fraxinus excelsior*, *Quercus* sp., and *Sorbus torminalis*). Analyses include the following tree species: *Abies alba*, *Acer pseudoplatanus*, *Carpinus betulus*, *Fagus sylvatica*, *Picea abies*, *Pinus sylvestris*, *Quercus* sp., *Fraxinus excelsior*, and *Sorbus torminalis*.

We tested whether we could deduce similar physiologically relevant drought thresholds solely from dendrometer signals, that is, MDS_norm_ and TWD_norm_. We found decreasing MDS_norm_ with increasing TWD_norm_ in most species (Fig. 4). We extracted TWD_norm_ at the point when MDS_norm_ = 0.5. The model for *F. excelsior*, *Quercus* sp., and *S. torminalis* did not reach MDS_norm_ = 0.5 (Fig. 4). The lowest TWD_norm_ at which MDS_norm_ = 0.5 was observed for *C. betulus* (TWD_norm_ = 2.04), followed by *F. sylvatica* (2.16), *A. pseudoplatanus* (2.48), *A. alba* (2.79), *P. abies* (3.61), and *P. sylvestris* (5.49) (Fig. 4). A similar ranking was found when analyzing data only from the dry year 2022 (Fig. S6). By contrast, during 2021, an exceptionally wet year at the site (Fig. S7), very few values exceeded a TWD_norm_ of 1. Consequently, it was not possible to establish an MDS_norm_ = 0.5 for any species under these conditions.

**Fig. 5:**
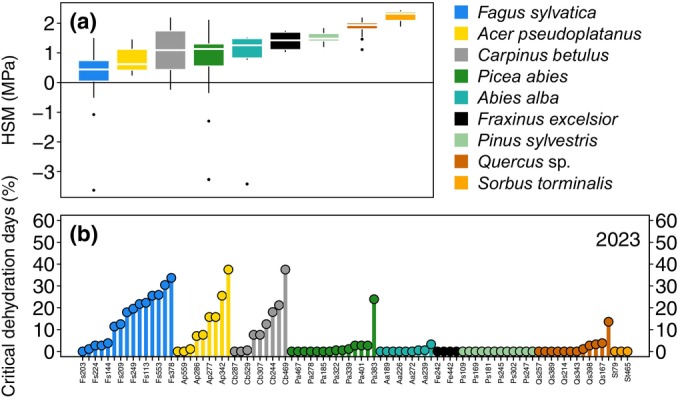
Vulnerability of monitored tree species based on leaf water potential (Ψ_leaf_) measurements and dendrometer signals. (a) Boxplots of the hydraulic safety margins (HSMs) for all species, considering the minimum Ψ_leaf_ recorded for each monitored tree and the point at which 50% of hydraulic conductivity is lost (*P*_50_). Boxplots show the median (line inside the box), the range between the 25^th^ and 75^th^ percentiles (box), and the smallest and largest values within 1.5 times that range (whiskers). Values outside this range are shown as individual points. (b) The percentage of leaf turgor loss days for each individual tree monitored in 2023. The species were ranked according to the ranking presented in (a). A critical dehydration day is defined as a day when the midday normalized tree water deficit (TWD_norm_) crosses the point at which TWD_norm_ crossed the MDS_norm_ = 0.5 threshold. The percentage is calculated based on the total number of days within the growing season from May to October. Each species is represented by a specific color, as shown in the legend. Analyses include the following tree species: *Abies alba*, *Acer pseudoplatanus*, *Carpinus betulus*, *Fagus sylvatica*, *Picea abies*, *Pinus sylvestris*, *Quercus* sp., *Fraxinus excelsior*, and *Sorbus torminalis*.

We then compared these TWD_norm_ thresholds, where MDS_norm_ = 0.5, with the linear fit at which the turgor loss point was reached in the pre‐dawn Ψ_leaf_ relationship with pre‐dawn TWD_norm_ (Fig. 3). This comparison revealed a small mean offset of 0.1 ± 0.6 pre‐dawn TWD_norm_ (Table 2) and showed a similar ranking between the two methods. However, note that for the broadleaved species it appears that MDS_norm_ 0.5 slightly overestimates the TWD_norm_ threshold, while for conifers it underestimated this value (Table 2). To evaluate the effectiveness of the TWD_norm_ threshold defined by MDS_norm_ = 0.5 in explaining hydraulic vulnerability, we quantified the number of days when these thresholds were reached during midday conditions over the entire monitoring period, which we termed ‘critical dehydration days’ (Fig. S8). This analysis, conducted for each individual tree, assessed whether the cumulative days, relative to the growing season, aligned with species‐specific HSM rankings. The HSMs revealed that *F. sylvatica* was closest to the point of 50% loss of xylem conductivity (*P*_50_), while *S. torminalis* was farthest (Fig. 5a). Similarly, the calculated leaf turgor loss days per tree corresponded closely with the HSM ranking (Fig. 5b). For *F. excelsior*, we substituted the *P*_50_ value of the ring‐porous *Quercus* sp. due to unavailable measurements for this species (Fig. 5).

**Table 2:** Overview of normalized tree water deficit (TWD_norm_) values at MDS_norm_ = 0.5 and turgor loss points.

Species	A: TWD_norm_ at MDS_norm_ = 0.5(−)	B: TWD_norm_ at turgor loss (−)	Delta (A−B)	MDS_norm_ at turgor loss (−)	Turgor loss point (MPa)
*Abies alba*	2.79	3.02	−0.22	0.46	−2.97
*Picea abies*	3.61	4.32	−0.71	0.40	−2.83
*Pinus sylvestris*	5.49	na	na	na	−2.55
*Carpinus betulus*	2.04	1.70	0.34	0.60	−2.78
*Fagus sylvatica*	2.17	1.60	0.57	0.67	−2.62
*Acer pseudoplatanus*	2.48	2.00	0.48	0.64	−2.11
*Fraxinus excelsior*	na	na	na	na	−3.44
*Quercus* sp.	na	3.22	na	0.63	−3.06
*Sorbus torminalis*	na	na	na	na	−3.18

This table provides an overview of the pre‐dawn TWD_norm_ values corresponding (from Fig. 3), and the turgor loss points (in MPa) as determined by the linear mixed‐effect model fit (presented in Fig. 4). ‘na’ is indicated where the fit did not cross the selected boundary or was not robust. Mean values are shown with SD (±).

## Discussion

We show that the combined use of normalized maximum daily shrinkage (MDS_norm_) and normalized pre‐dawn tree water deficit (TWD_norm_) dynamics provides critical insights into the drought stress status of individual trees. Our findings advance the application of dendrometer measurements beyond growth‐pattern analyses (Etzold *et al*., 2022; Tumajer *et al*., 2022) and wood phenology studies (Deslauriers *et al*., 2007; van der Maaten *et al*., 2018). Furthermore, the findings extend dendrometer Ψ_leaf_ studies (Dietrich *et al*., 2018; Walthert *et al*., 2024) and highlight their value in providing critical information on tree hydraulics (Zweifel *et al*., 2007). We found evidence that supports our hypothesized framework (Fig. 1), where we suggested a robust TWD_norm_ threshold past which trees are likely to lose leaf turgor (Fig. 3), helping to identify drought‐vulnerable species and vulnerable individual trees. We therefore emphasize the importance of applying TWD_norm_ to quantify drought stress, which is crucial for making informed forest management decisions in the face of climate change (Zweifel *et al*., 2023).

### Signals of stomatal closure in the diel pattern of stem shrinkage and swelling

Across the studied conifer and broadleaf tree species, we observed that *g*_s_ was related to TWD_norm_, with low *g*_s_ especially when TWD surpassed MDS_max_ (TWD_norm_ > 1, Fig. 2a). This finding aligns with our first hypothesis (H1), supporting that we can disentangle when mature trees use stomatal control to mitigate the excessive daily reduction of water reserves in the storage tissues, while under well‐hydrated conditions allowing the MDS to reach its maximum. We hypothesize that this control is optimized to avoid excessively negative water potentials in the stem, which could impede the rehydration necessary for maintaining turgor in growing stem tissues (Peters *et al*., 2023, 2025; Potkay & Feng, 2023). However, while all species exhibited a consistent reduction in *g*_s_ with TWD_norm_, there was still ample variability around the model fits (Fig. 2), which could be attributed to several factors: (1) handling of the LI‐COR and measurement inaccuracies (Busch *et al*., 2024); (2) the possibility that single leaf‐level measurements may not fully represent whole‐tree dynamics, as captured by dendrometers (see Mencuccini *et al*., 2019 for upscaling challenges); or (3) measurements being taken earlier in the day than when the point of full stomatal closure was reached (see diurnal dynamics in Zweifel *et al*., 2001).

Our findings align with a long‐standing recognition that tree stems undergo diurnal shrinkage and swelling due to internal water dynamics – a phenomenon documented as early as the 1960s (Kozlowski & Winget, 1964). The use of automated dendrometers, which have become increasingly widespread, has significantly enhanced our ability to monitor these patterns in high temporal resolution. This technological advancement has deepened our understanding of tree water use and its variability across species and environmental conditions (Klepper *et al*., 1971; Steppe *et al*., 2006; Verbeeck *et al*., 2007; Zweifel *et al*., 2007; De Swaef *et al*., 2015). In this context, numerous studies have demonstrated statistical links between MDS and tree hydraulic mechanisms (King *et al*., 2013; Ježík *et al*., 2015; Herrmann *et al*., 2016; Schäfer *et al*., 2018; Tian *et al*., 2019). However, direct empirical evidence of the tight coupling between TWD_norm_, MDS_norm_, and *g*_s_ in tall, mature trees, as shown here, has been lacking. Our findings highlight MDS_max_ as an ecophysiologically relevant threshold for normalizing TWD time series to TWD_norm_, offering a species‐specific and meaningful standardization point. Nonetheless, the structural traits driving variability in MDS_max_ patterns, such as phloem density or the proportion of living to dead bark, remain uncertain and warrant further investigation.

### Drought stress occurrence at a uniform water deficit within a species after normalizing TWD

In H2, we proposed that species will show a species‐specific TWD_norm_ above 1 at which the turgor loss point is reached, which can explain drought vulnerability. We found that indeed there is a robust TWD_norm_ value per species that indicates ψ_leaf_ values at, or close to, turgor loss (Fig. 3). This is a critical finding, as scaling TWD_norm_ in relation to the leaf turgor loss point (in MPa) could help to assess how close a species is to leaf wilting (Bartlett *et al*., 2012b), a point occurring before the formation of lethal embolisms which damages the hydraulic architecture (Choat *et al*., 2016; Arend *et al*., 2021; Walthert *et al*., 2021). These results provide evidence for the effectiveness of detecting drought stress by using TWD_norm_ thresholds without needing continuous ψ_leaf_ measurements.

Normalized TWD effectively reduced the variability in absolute TWD between trees, enabling us to compare trees and species, and allowed us to identify robust and species‐specific drought stress thresholds. In other studies, normalizing TWD to reduce variance has typically involved standardizing dry years against relatively wetter years (Salomón *et al*., 2022) or using the maximum TWD values recorded (Dietrich *et al*., 2018). Both methods, however, depend on the occurrence of specific conditions (wet vs dry yr), which can limit their applicability. By contrast, TWD_norm_ is less constrained by these limitations, as it is more likely that maximum transpiration and stomatal control will occur at one point across a few growing seasons (see Fig. S4; Flo *et al*., 2021). This robustness makes the TWD_norm_ method more reliable, as demonstrated previously, where pre‐dawn TWD_norm_ provided crucial insights into the rehydration dynamics of different temperate tree species (Peters *et al*., 2023).

### Relevance of reduced daily shrinkage with increasing TWD

Daily stem shrinkage reflects diurnal imbalances between water input through root uptake and output via transpiration. These dynamics result in stem diameter changes that are buffered by water release from elastic tissues. As such, shrinkage amplitude tends to increase under conditions of high evaporative demand, sufficient internal water storage, and limited soil water supply (Zweifel *et al*., 2001; Steppe *et al*., 2006). However, similar shrinkage patterns may arise from different combinations of these drivers, which emphasizes the need for contextual interpretation of shrinking dynamics. Our boundary‐line analysis revealed that some species reduced their MDS_norm_ below 0.5, indicating a transition toward drought stress (Fig. 5). In this range, species‐specific rankings may provide valuable insights into physiological drought sensitivity. However, note that the 50% threshold is an approximation, as the MDS_norm_ at turgor loss did deviate (with a range of *c*. 0.2), where broadleaved species showed turgor loss at higher MDS_norm_, while conifers showed this point at lower MDS_norm_ (Table 2). The reduction in MDS_norm_ with increasing TWD_norm_ likely reflects a combination of stomatal closure, reduced root water uptake, and compressibility of the elastic storage tissues, which limits further shrinkage (Peters *et al*., 2021). By contrast, under well‐hydrated conditions, water release from elastic tissues contributes more to transpiration, resulting in greater daily shrinkage (Zweifel & Häsler, 2001; De Schepper *et al*., 2012). Notably, *F. excelsior*, *Quercus* sp., and *S. torminalis* did not appear to reach this dehydration threshold during the study period, suggesting they did not experience severe drought stress. We focused on pre‐dawn TWD_norm_ due to its closer relationship with rehydration status and its relative insensitivity to vapor pressure deficit variability (Salomón *et al*., 2022), though a similar species ranking was obtained using midday TWD_norm_ (Fig. S5).

In our study, *A. alba*, *P. abies*, and *F. sylvatica* crossed the 50% hydraulic conductivity loss threshold (*P*_50_; Fig. S5), allowing us to test species‐specific HSMs (Meinzer *et al*., 2009) against TWD_norm_ (Fig. 5). While ψ_leaf_ responses are not linear over time (Arend *et al*., 2021), we found that HSM rankings (Fig. 5a), identifying *F. sylvatica* as the most vulnerable species, corresponded well with the cumulative days when TWD_norm_ exceeded the threshold where MDS_norm_ equals 0.5 (Fig. 5b). This aligns with previous studies reporting high drought vulnerability in *F. sylvatica* (Schuldt *et al*., 2020) and *P. abies* (Gharun *et al*., 2020; Trotsiuk *et al*., 2020), linked to their reliance on shallow root water uptake (Brinkmann *et al*., 2019; Kahmen *et al*., 2022). By contrast, deeper‐rooting species such as *S. torminalis*, *Quercus* sp., and *F. excelsior* are better equipped to withstand drought, while *P. sylvestris* exhibits drought resistance through water‐saving strategies (Peters *et al*., 2023). These results highlight the potential of dendrometer signals to determine both the timing and intensity of drought stress across tree species.

### Critical considerations on the novel method and future perspective

Establishing critical TWD_norm_ thresholds requires careful consideration, as various processing decisions and data characteristics can significantly influence the point at which MDS_norm_ = 0.5 is reached. It is crucial to have sufficient data points covering a wide TWD_norm_ range, especially TWD_norm_ > 1, as models like GAMM can become unreliable with sparse data (see Figs S6, S7). Meticulous curation of stem radius measurements is also essential, as errors can substantially impact the TWD time series (Knüsel *et al*., 2021). Selecting the appropriate growing season window is important, as winter shrinkage due to frost can impair analyses (Zweifel *et al*., 2000; Zweifel & Häsler, 2000). Moreover, for obtaining a stable MDS_max_ value, it is advised to have at least 2–3 yr of dendrometer data (see Fig. S4). Although this study uniquely uses extensive ecophysiological measurements, similar tests could be conducted at other sites to validate our methodology (e.g. Dietrich *et al*., 2019). Moreover, an important step forward would be to mechanistically explain the relationship between TWD and ψ_leaf_, where existing mechanistic models could provide an important theoretical perspective (Zweifel *et al*., 2007; Peters *et al*., 2021).

While our results reveal clear species‐specific patterns, some uncertainties in TWD measurements and hydraulic traits must be acknowledged. Factors such as minor sensor shifts can artifactually change TWD values. Additionally, removing dead bark during dendrometer installation, and over longer monitoring periods, is necessary to prevent hygroscopic swelling, which could also impact TWD measurements of species with thick outer bark (i.e. *Picea abies*, *Pinus sylvestris*). Moreover, intra‐tree variability in tissue properties can affect TWD interpretation at low water potentials. The unusually low TWD_norm_ values for *A. alba* in Fig. 3, for example, reflect a tree that surpassed its cavitation threshold and subsequently died, rather than a typical physiological response. As such, we do not know how TWD_norm_ behaves after trees have reached water potentials at which the hydraulic architecture is severely damaged (i.e. Arend *et al*., 2022). As such, the normalization of TWD and MDS might not apply to trees undergoing strong physiological decline, such as those experiencing crown dieback or loss of leaf area. In these cases, the normalization might require a dynamic adjustment over time. These points underscore the need for careful data curation and interpretation.

Developing standardized, field‐based metrics such as TWD_norm_ is crucial for interpreting continuous dendrometer data in ecophysiological studies (i.e. Peters *et al*., 2023). While *ex situ* laboratory‐derived traits like turgor loss point and xylem vulnerability (*P*_50_) provide valuable insight into plant hydraulic properties, they may not fully capture the dynamic responses of trees to environmental stressors *in situ*. Field‐based metrics offer a complementary perspective, enabling the assessment of tree water status under varying conditions of vapor pressure deficit and soil moisture (see Mencuccini *et al*., 2024 for Ψ_leaf_ dynamics). This approach aligns with recent efforts to develop plant‐centric water stress metrics that integrate environmental drives and physiological response (i.e. Flo *et al*., 2021), enhancing our understanding of plant water use strategies in natural settings.

Our analyses provide a valuable method for using MDS_norm_ and TWD_norm_ as a benchmark to detect drought stress, paving the way for leveraging dendrometers to better understand the impact of climate change on forest vitality (Zweifel *et al*., 2023). With the increasing demand for on‐the‐ground data on tree physiological processes to feed modeling (Babst *et al*., 2021), further exploration of automated dendrometer signals will be crucial. Systematic assessments of MDS_max_ across different sites and species could enhance the applicability of TWD_norm_ (e.g. Pérez‐López *et al*., 2013). Additionally, using this method on a wide range of tree species and ecosystems, including tropical forests (De Mil *et al*., 2019; Peters *et al*., 2023; Zhou *et al*., 2023), would further confirm the broader utility of this novel approach.

## Competing interests

None declared.

## Disclaimer

The New Phytologist Foundation remains neutral with regard to jurisdictional claims in maps and in any institutional affiliations.

## Supporting information


**Fig. S1** Common point dendrometer signals.
**Fig. S2** Relationship between normalized pre‐dawn tree water deficit (TWD_norm_), maximum daily shrinkage (MDS_norm_), and normalized midday stomatal conductance (*g*_s_/*g*_s.max_) over all species.
**Fig. S3** Relationship between pre‐dawn normalized tree water deficit (TWD_norm_) and midday stomatal conductance (*g*_s_).
**Fig. S4** Variance of normalized maximum daily shrinkage (MDS_norm_) between the entire time series and the specific number of years (ΔMDS_norm_).
**Fig. S5** Relationship between pre‐dawn leaf water potential (Ψ_leaf_) and pre‐dawn normalized tree water deficit (TWD_norm_).
**Fig. S6** Response of normalized maximum daily shrinkage (MDS_norm_) to increasing pre‐dawn normalized tree water deficit (TWD_norm_) for 2022, a year considered dry at the research site.
**Fig. S7** Response of normalized maximum daily shrinkage (MDS_norm_) to increasing pre‐dawn normalized tree water deficit (TWD_norm_) for 2021, a year considered wet at the research site.
**Fig. S8** Time series of midday normalized tree water deficit (TWD_norm_) in 2023. This figure presents the average species‐specific TWD_norm_ and confidence intervals for 2023.
**Table S1** Description of our model describing the relationship between normalized pre‐dawn tree water deficit (TWD_norm_), normalized maximum daily shrinkage (MDS_norm_), and stomatal conductance (*g*_s_) across species.
Please note: Wiley is not responsible for the content or functionality of any Supporting Information supplied by the authors. Any queries (other than missing material) should be directed to the *New Phytologist* Central Office.

## Data Availability

The gas exchange, dendrometer, and leaf water potential data used in this study are available via Zenodo at https://zenodo.org/records/15463588.
