# Supplementary material for: Normalized tree water deficit: an automated dendrometer signal to quantify drought stress in trees
Source: New Phytol. 2025 Jun 11;247(3):1186–98. doi: 10.1111/nph.70266 (PMC12222926; doi:10.1111/nph.70266)
Supplement: Supplementary file 1 — Fig. S1 Common point dendrometer signals. Fig. S2 Relationship between normalized pre‐dawn tree water deficit (TWDnorm), maximum daily shrinkage (MDSnorm), and normalized midday stomatal conductance (g s/g s.max) over all species. Fig. S3 Relationship between pre‐dawn normalized tree water deficit (TWDnorm) and midday stomatal conductance (g s). Fig. S4 Variance of normalized maximum daily shrinkage (MDSnorm) between the entire time series and the specific number of years (ΔMDSnorm). Fig. S5 Relationship between pre‐dawn leaf water potential (Ψleaf) and pre‐dawn normalized tree water deficit (TWDnorm). Fig. S6 Response of normalized maximum daily shrinkage (MDSnorm) to increasing pre‐dawn normalized tree water deficit (TWDnorm) for 2022, a year considered dry at the research site. Fig. S7 Response of normalized maximum daily shrinkage (MDSnorm) to increasing pre‐dawn normalized tree water deficit (TWDnorm) for 2021, a year considered wet at the research site. Fig. S8 Time series of midday normalized tree water deficit (TWDnorm) in 2023. This figure presents the average species‐specific TWDnorm and confidence intervals for 2023. Table S1 Description of our model describing the relationship between normalized pre‐dawn tree water deficit (TWDnorm), normalized maximum daily shrinkage (MDSnorm), and stomatal conductance (g s) across species. Please note: Wiley is not responsible for the content or functionality of any Supporting Information supplied by the authors. Any queries (other than missing material) should be directed to the New Phytologist Central Office. [file NPH-247-1186-s001.docx]

**New Phytologist Supplementary Information:**

Article title: Normalized tree water deficit: An automated dendrometer signal to quantify drought stress in trees

Authors: Richard L. Peters, David Basler, Roman Zweifel, David N. Steger, Tobias Zhorzel, Cedric Zahnd, Günter Hoch, Ansgar Kahmen

Article acceptance date: 19 May 2025


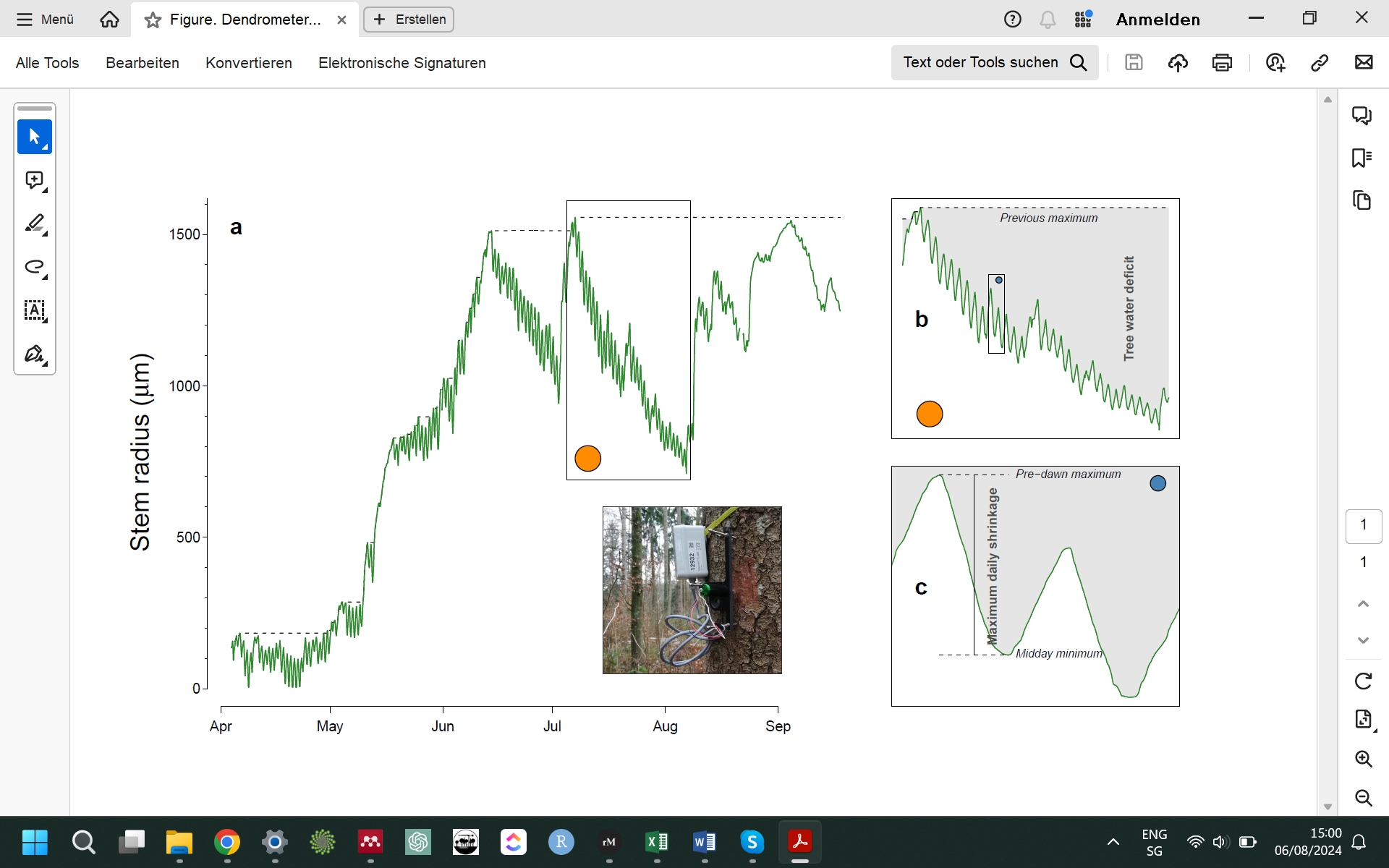


**Figure S1.** Common point dendrometer signals. (a) Timeseries of dendrometer measurements showing stem radius changes for a *Picea abies* tree at the Swiss Canopy Crane II (SCCII) site in 2018. The coloured line represents the raw data, while the dashed line indicates the running maximum radius. The orange dot within the box marks a period of excessive shrinkage, which is further detailed in panel (b). (b) A close-up of the highlighted period, illustrating the tree water deficit (TWD) as the grey area between the previous maximum radius and the raw measurements. The box outlines a two-day period, further examined in panel (c). (c) Two diurnal cycles showing stem swelling in the morning and stem shrinkage during midday. The difference between the maximum morning swelling and the minimum midday shrinkage is defined as the maximum daily shrinkage (MDS).


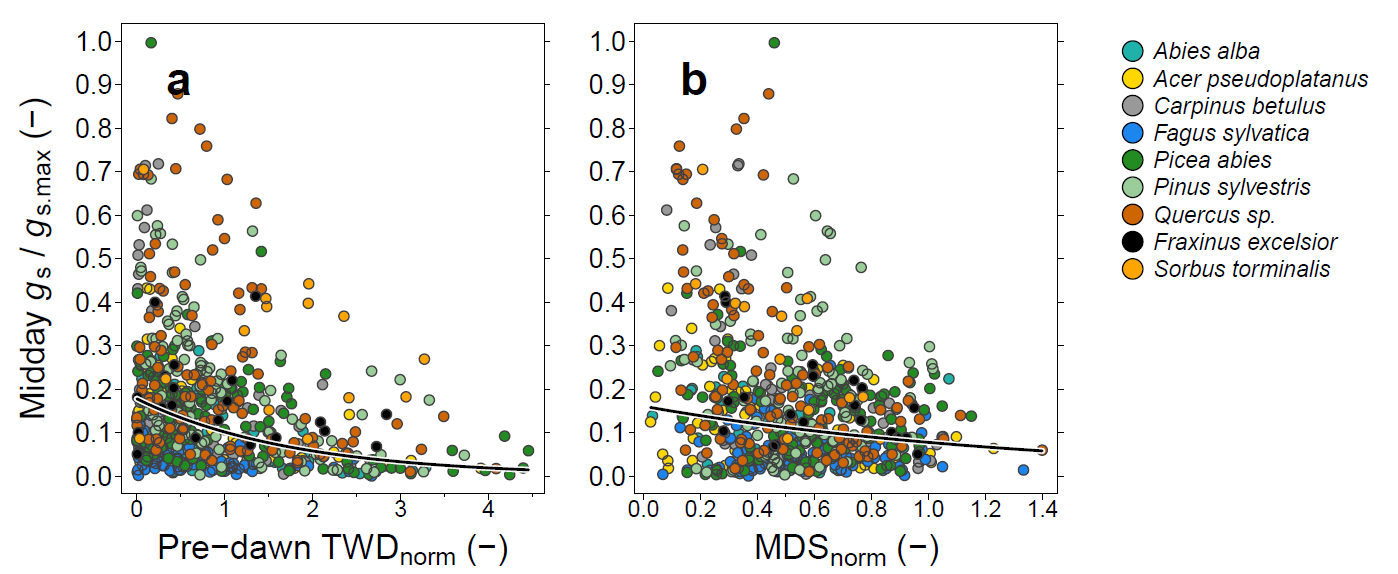


**Figure S2.** Relationship between normalized pre-dawn tree water deficit (TWD_norm_), maximum daily shrinkage (MDS_norm_), and normalized midday stomatal conductance (*g*_s_ / *g*_s.max_) over all species. The normalization was performed by dividing the *g*_s_ by the species-specific maximum *g*_s_. A linear mixed-effect model was fitted to the data (with tree nested within species as a random effect). A linear mixed-effect model was fitted on the data (random effect = random intercept of tree nested in species), with the solid black line showing the mean. Within the model pre-dawn TWD_norm_ and MDS_norm_ are interacting independent variables to explain midday *g*_s_. The colours correspond to different species, as shown in the legend.


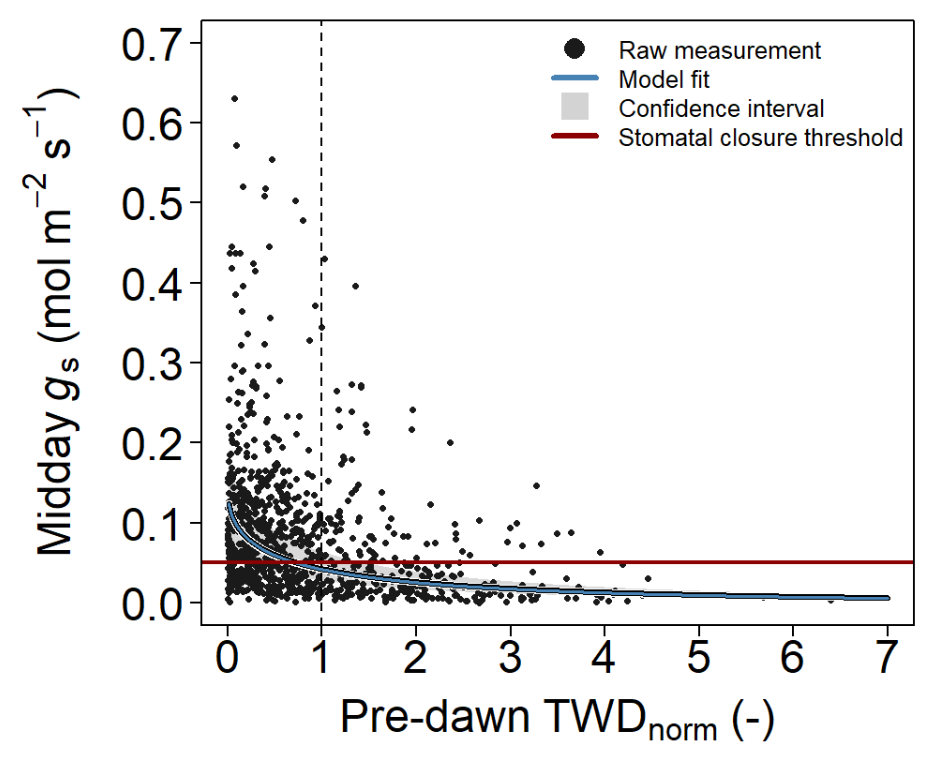


**Figure S3.** Relationship between pre-dawn normalized tree water deficit (TWD_norm_) and midday stomatal conductance (*g*_s_). This figure shows the back-transformed linear relationship between square-root-transformed pre-dawn TWD_norm_ and log-transformed *g*_s_ values. A linear mixed-effect model was fitted to the data, with tree nested within species as a random effect. The red line indicates the 0.05 mol m^-2^ s^-1^ *g*_s_ threshold, commonly considered the point of stomatal closure (Xu et al. 2022).


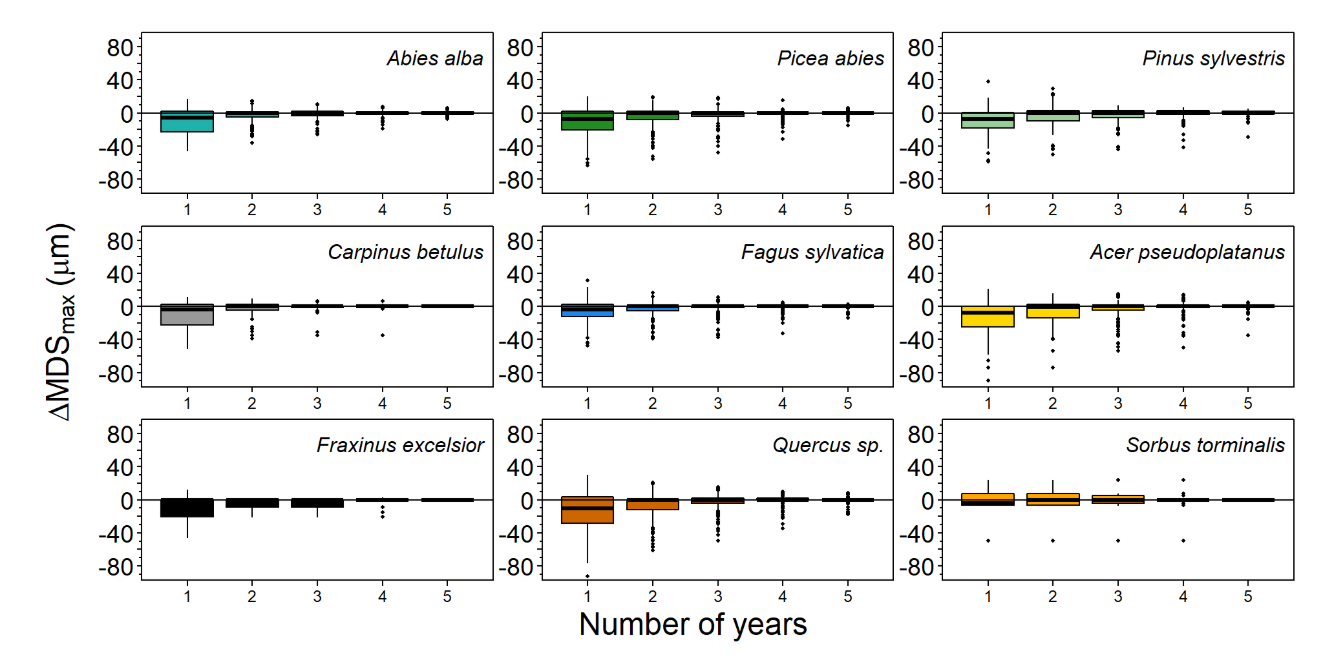


**Figure S4.** Variance of normalized maximum daily shrinkage (MDS_norm_) between the entire time series and specific number of years (ΔMDS_norm_). Boxplots are provided for each species, which include all monitored tree per species. The boxplot shows the median (line inside the box), interquartile range (IQR; box), whiskers (1.5 × IQR), and outliers (dots beyond the whiskers). For each tree 1000 resamplings were performed (without replacement) and compared to the MDS/MDS_max_ of the entire series. From the analyses with one year of data we strongly underestimate the actual MDS/MDS_max_ in our ecosystem.

**
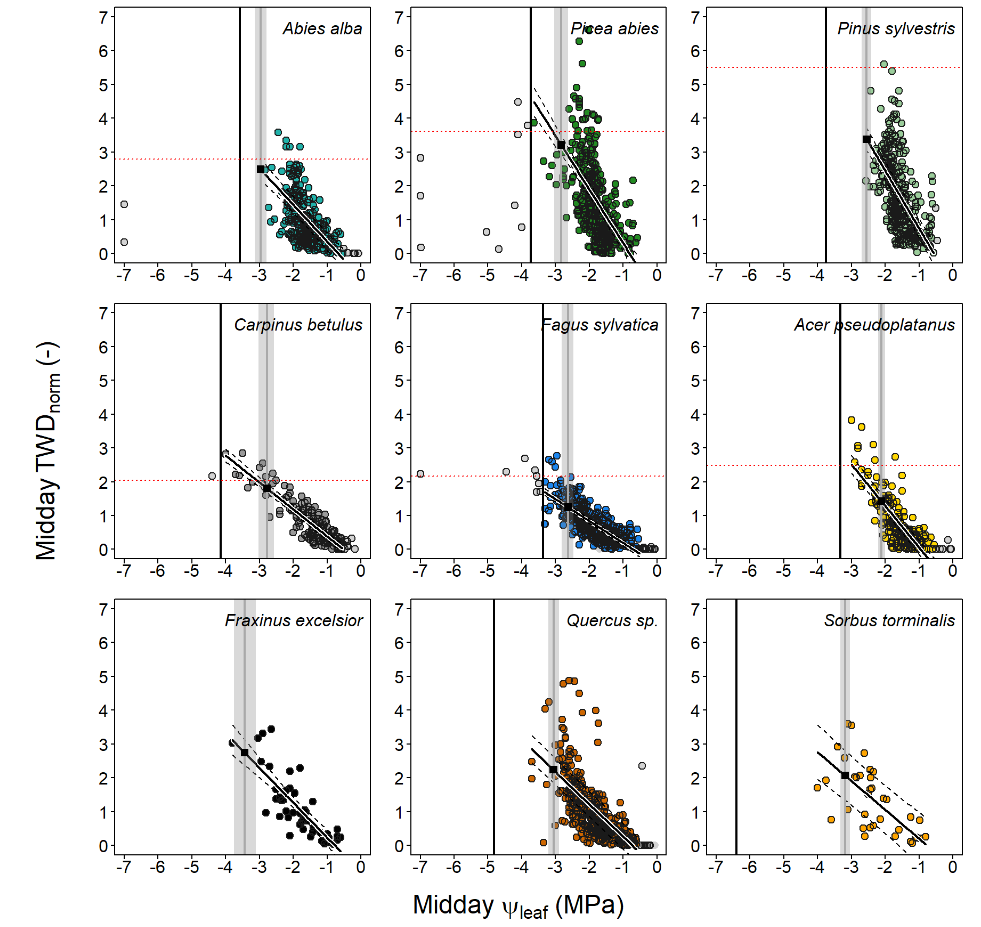
**

**Figure S5.** Relationship between pre-dawn leaf water potential (*Ψ*_leaf_) and pre-dawn normalized tree water deficit (TWD_norm_). The TWD_norm_ at which MDS_norm_ = 0.5 is marked with a red dashed line for reference. Note that no standardized dehydration points were found for *F. excelsior*, *Quercus sp.*, and *S. torminalis*. Critical ecophysiological thresholds presented include the turgor loss point (solid grey line with the upper and lower quantile as shading) and the point of 50% loss of xylem conductivity (vertical black line). Zero TWD_norm_ values are indicated by grey dots. Also, *Ψ*_leaf_ values below -0.5 MPa and above the point of 50% loss of xylem conductivity are presented as circles and not included in the analysis to ensure linearity. A linear mixed-effect model was fitted to illustrate the species-specific behavior (bold black line), including the confidence interval (dashed lines).

**
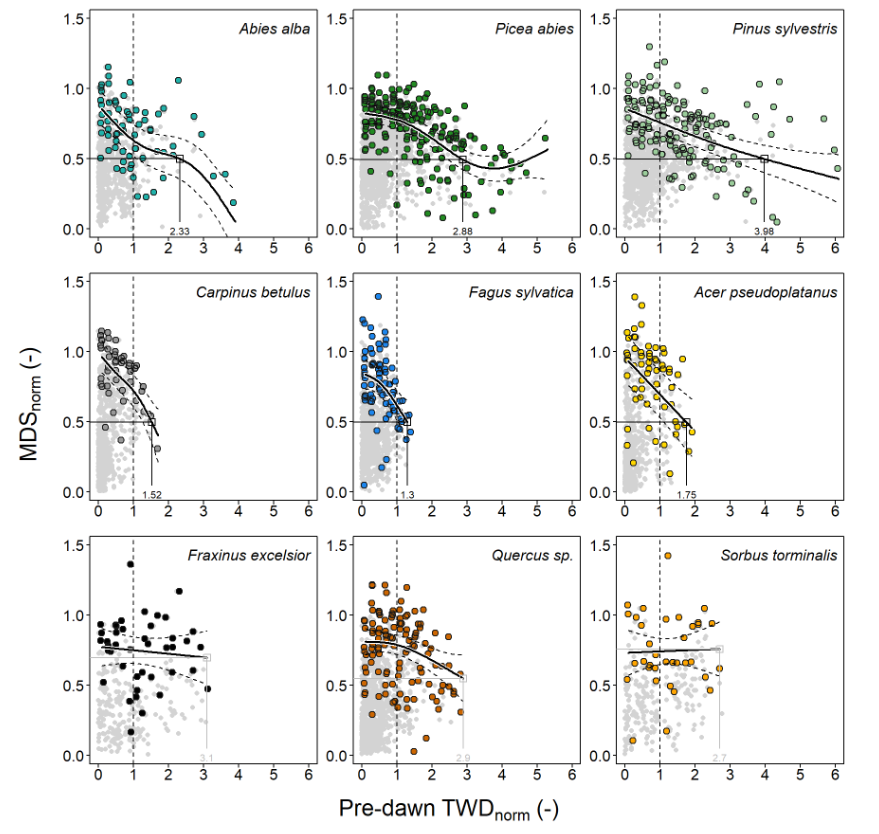
**

**Figure S6.** Response of normalized maximum daily shrinkage (MDS_norm_) to increasing pre-dawn normalized tree water deficit (TWD_norm_) for 2022, a year considered dry at the research site. Maximum values across fixed-size TWD_norm_ bins were determined for each tree (colored dots) from the raw measurements (grey dots). A generalized additive mixed-effect model (GAMM) was fitted to illustrate the species-specific behavior (bold black line), with the confidence interval represented by dashed lines. The number in each panel represents the point at an MDS_norm_ of 0.5. If a normalized MDS of 0.5 was never reached by the GAMM, the maximum value is presented in grey (e.g., for *F. excelsior*, *Quercus sp*., and *S. torminalis*).


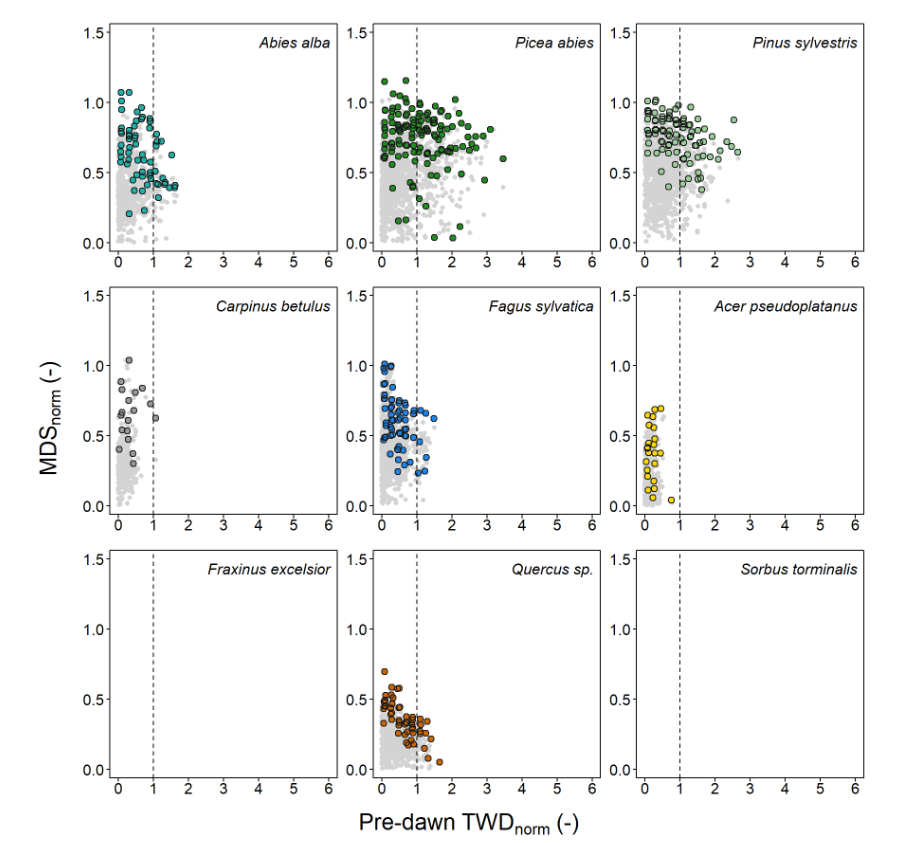


**Figure S7.** Response of normalized maximum daily shrinkage (MDS_norm_) to increasing pre-dawn normalized tree water deficit (TWD_norm_) for 2021, a year considered wet at the research site. Maximum values across fixed-size TWD_norm_ bins were determined for each tree (colored dots) from the raw measurements (grey dots). Very few points exceeded pre-dawn TWD_norm_ = 1, making it impossible to fit a robust generalized additive mixed-effect model (GAMM). Note that no point dendrometer data was recorded for *F. excelsior* and *S. torminalis* during this year.

**
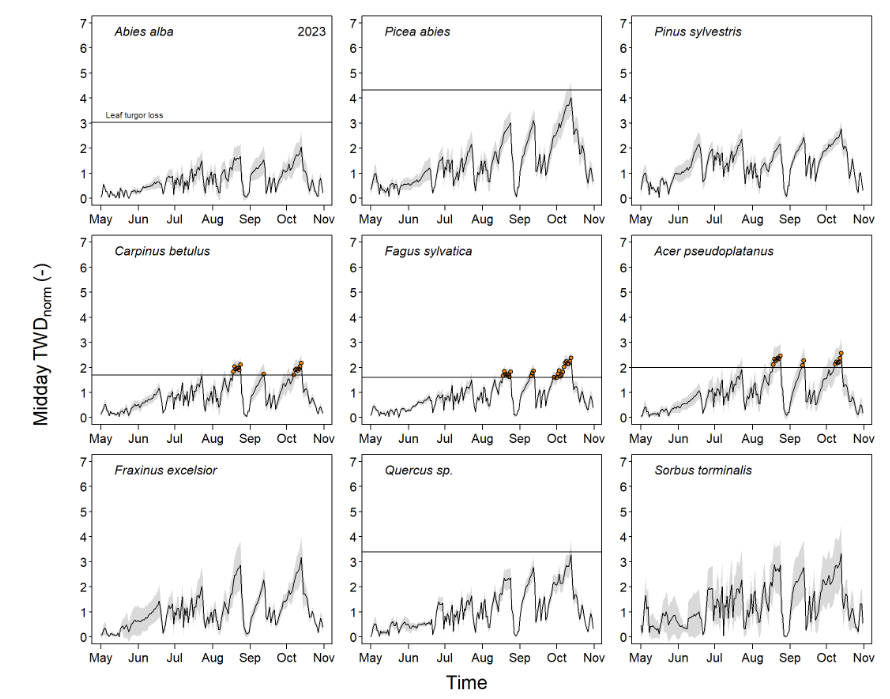
**

**Figure S8.** Time-series of midday normalized tree water deficit (TWD_norm_) in 2023. This figure presents the average species-specific TWD_norm_ and confidence intervals for 2023. The solid line marks the TWDnorm at leaf turgor loss.

**Table S1.** Description of our model describing the relationship between normalized pre-dawn tree water deficit (TWD_norm_), normalized maximum daily shrinkage (MDS_norm_), and stomatal conductance (*g*_s_) across species. Here the tree was nested in the species as random intercepts. The independent variables were scaled for comparison. We provide the model estimate for each parameter, the standard error, the degrees of freedom (DF), the t-value, and the *P*-value calculation.

| **Parameter** | **Estimate** | **St. Error** | **DF** | **t-value** | ***P*-value** |
| --- | --- | --- | --- | --- | --- |
| ß_0_ (intercept) | -2.88 | 0.21 | 591 | -13.93 | < 0.0001 |
| ß_MDSnorm_ (scaled) | -0.18 | 0.03 |  | -6.26 | < 0.0001 |
| ß_TWDnorm_ (scaled) | -0.48 | 0.03 |  | -14.81 | < 0.0001 |
| ß_MDSnorm_ : ß_TWDnorm_ | 0.12 | 0.03 |  | 3.81 | 0.0002 |
